# Xanthine Oxidase Inhibitors from *Filipendula ulmaria* (L.) Maxim. and Their Efficient Detections by HPTLC and HPLC Analyses

**DOI:** 10.3390/molecules26071939

**Published:** 2021-03-30

**Authors:** Maël Gainche, Clémence Ogeron, Isabelle Ripoche, François Senejoux, Juliette Cholet, Caroline Decombat, Laetitia Delort, Jean-Yves Berthon, Etienne Saunier, Florence Caldefie Chezet, Pierre Chalard

**Affiliations:** 1 Clermont Auvergne INP, Université Clermont Auvergne, CNRS, ICCF, F-63000 Clermont-Ferrand, France; isabelle.ripoche@sigma-clermont.fr (I.R.); pierre.chalard@sigma-clermont.fr (P.C.); 2 Université Clermont-Auvergne, INRA, UNH, Unité de Nutrition Humaine, CRNH Auvergne, F-63000 Clermont-Ferrand, France; clemence.ogeron@uca.fr (C.O.); francois.senejoux@uca.fr (F.S.); juliette.cholet@uca.fr (J.C.); Caroline.DECOMBAT@uca.fr (C.D.); laetitia.delort@uca.fr (L.D.); florence.caldefie-chezet@uca.fr (F.C.C.); 3 Greentech, Biopôle Clermont-Limagne, 63360 Saint-Beauzire, France; jeanyvesberthon@greentech.fr; 4 Dômes Pharma, 3 Rue André Citroën, 63430 Pont-du-Château, France; e.saunier@domespharma.com

**Keywords:** *Filipendula ulmaria*, meadowsweet, xanthine oxidase, flavonoids, HPTLC bioautography, HPLC, natural products

## Abstract

*Filipendula ulmaria* is a plant commonly used for the treatment of several pathologies, such as diarrhoea, ulcers, pain, stomach aches, fevers, and gout. Our study focused on the use of *F. ulmaria* for the treatment of gout disease. We first studied the chemical composition of a methanolic extract of the aerial parts and demonstrated its xanthine oxidase (XO) inhibitory activity. Then, we performed a fractionation and evaluated the most XO inhibitory active fractions by UV measurement. Purification of some fractions allowed the determination of the inhibitory activity of pure compounds. We demonstrated that spiraeoside, a glycosylated flavonoid, possesses an activity around 25 times higher than allopurinol, used as a reference in the treatment of gout disease. In order to easily and quickly identify potent inhibitors in complex matrix, we developed a complementary strategy based on an HPLC method and an Effect Directed Assay (EDA) method combining HPTLC and biochemical assays. The HPLC method, capable of determining compounds exhibiting interactions with the enzyme, could be an efficient strategy for evaluating potent enzyme inhibitors in a complex mixture. This strategy could be applied for quantitative assays using LC/MS experiments.

## 1. Introduction

*Filipendula ulmaria* (L.) Maxim. (formerly called *Spiraea ulmaria*), also known as meadowsweet, is a very common and widespread herbaceous perennial plant belonging to the Rosaceae family. The uses of this species were highlighted by several ethnopharmacological surveys across Europe, mostly for its sweet flavor and its numerous biological activities [1,2]. In traditional uses, its leaves and flowers are used in decoction for their flavoring capacity, as well as for the treatment of various pathologies, such as rheumatism, gout, headache, or pneumonia [2,3].

Several studies demonstrated very promising antiproliferative and anti-inflammatory properties, mostly due to its content in salicylic derivatives (salicylic acid, methylsalycylate, etc.) [4,5,6]. In addition, phytochemical studies showed the presence of two other important classes of compounds: flavonoids, such as quercetin and kaempferol derivatives (hyperoside, isoquercitrin, rutoside, spiraeoside, astragalin), and tannins (tellimagrandin I and II, rugosin A, B, D, E) [7,8,9].

Most of the phytochemical and biological surveys on *F. ulmaria* were linked to its uses against rheumatism, but none of them were focused on its use for gout. Gout is a disease closely linked to the formation of monosodium urate crystals in tissues, mostly in joints [10]. Those urate crystals, due to a high concentration of uric acid in the blood (hyperuricemia), are associated to an enzyme in the human body, xanthine oxidase (XO). This key enzyme, belonging to the molybdenum hydroxylase superfamily, catalyzes the oxidation of hypoxanthine to xanthine and then to uric acid. This transformation produces a large amount of reactive oxygen species (ROS), as subproducts, such as H_2_O_2_ or O_2_^−.^ [11].

To treat gout, two main strategies are currently used. The first one consists of increasing the excretion of uric acid in order to lower its level in serum, and to prevent the formation of urate crystals, generally with drugs such as probenicid. The second way is a combined action of anti-inflammatory drugs and a XO inhibitors, such as allopurinol or febuxostat [12]. Most of the time, allopurinol is the first-intention treatment, but recent studies linked high doses of allopurinol with a prevalence of allergic reactions [13]. Nowadays, the development of new XO inhibitors focuses mainly on compounds belonging to the flavonoids family, and very promising results were obtained with compounds such as quercetin or luteolin derivatives [14,15,16].

The aim of this study was to characterize the *F. ulmaria* aerial parts extract and to determine the potential XO inhibitors present in the extract using quick and efficient analytical methods, such as HPLC and HPTLC Effect Directed Analysis (EDA). An EDA HPTLC strategy has become, over the last decades, one of the most efficient screening methods for the identification of bioactive compounds in a complex matrix, combining planar chromatography and chemical/biological/biochemical analyses. This recent strategy has been developed to specifically facilitate the bio/chemical-bioguided fractionation of plant extracts.

One of the most common EDA methods combining HPTLC and chemical assays consists of evaluating the antioxidant activity of plant extracts using a radical scavenging activity assay (against DDPH*) [17]. The compatibility of HPTLC with microbial and biochemical assays permits an evaluation directly on TLC plates of the antifungal and antibacterial activities of compounds present in complex mixtures. Recently, a particular effort has been made in order to determine the inhibitory activities on several enzymes, such as glucosidase, amylase, and tyrosinase [18].

In order to develop and validate our methods, we first performed a bioguided fractionation to identify the active compounds by UV spectrophotometer measurements, and then confirmed the efficiency of the developed analytical methods on the crude extract to quickly identify the secondary metabolites exhibiting XO inhibition.

## 2. Results and Discussion

### 2.1. Phytochemical Profile of F. ulmaria Aerial Parts Methanolic Extract

**Figure 1:**
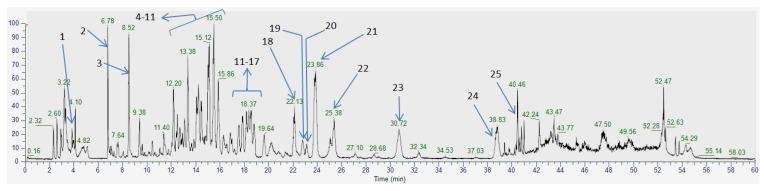
Chromatogram with compounds identified in *F. ulmaria* aerial parts.

The phytochemical profile of the crude extract, performed by HPLC-MS, was in agreement with previous studies. The extract contained mainly glycosylated flavonoids derived from quercetin **24** (rutoside **12**, isoquercitrin **14**, hyperoside **16**, miquelianin **17**, spiraeoside **21**), kaempferol **25** (astragalin **18**, astragalin-2″-O-gallate **19**, kaempferol-4′-O-glucoside **22**) and rhamnetin (isorhamnetin-O-hexoside **20**). The presence of tannins (tellimagrandin I **7** and II **10**, rugosin B **5**, A **9**, D **11** and E **8**), catechin **6,** and phenolic acids (gallic acid **3,** chlorogenic acid **4**, ellagic acid **13** and salicylic acid **23**) was also evaluated [7,19,20,21]. As previously described, it seems that the most abundant compounds in the extract are tellimagrandin II, rugosin D, and spiraeoside (Figure 1, Table 1) [8,20].

To confirm the XO inhibitory activity of the crude extract, we measured the IC_50_ values using a described UV spectroscopy method [22]. The crude extract showed a significant inhibitory activity with an IC_50_ of 8.3 ± 0.3 µg/mL compared to the activity of allopurinol (IC_50_ = 2.9 ± 0.1 µg/mL). In order to evaluate the potential activity of each compound present in the extract, we performed a liquid/liquid partition and purification of some metabolites to evaluate their XO inhibitory activities.

### 2.2. Liquid/Liquid Partition

In order to investigate the XO inhibitory activity of *F. ulmaria*, aerial parts extract was submitted to a bioguided fractionation. The crude methanolic extract was engaged in a liquid/liquid partition successively, with *n*-hexane, dichloromethane (CH_2_Cl_2_), ethyl acetate (EtOAc), *n*-butanol (*n*-BuOH), and water (Supplementary Material Figure S1). For each fraction, the XO inhibition activity was evaluated, and the chemical profile was identified.

If *n*-hexane, CH_2_Cl_2_, and water fractions did not exhibit any activity with IC_50_ > 100 µg/mL, EtOAc and *n*-BuOH fractions possess a good inhibitory activity with low IC_50_ values (respectively, IC_50_ 2.6 ± 0.1 µg/mL and 12.3 ± 1.2 µg/mL). The IC_50_ value of the EtOAc fraction is quite similar to the IC_50_ value observed for allopurinol.

The study of the chemical profiles of both *n*-BuOH and EtOAc fractions show a relatively similar composition with mainly flavonoids and tannins, but in higher concentrations in the EtOAc fraction. This difference of composition could explain the slight differences of activity between those two fractions. The inhibitory activity could be linked to the presence of compounds, such as tellimagrandin II, spiraeoside, quercetin, or kaempferol, present in high quantities in the EtOAc fraction.

Considering the promising activity of the EtOAc fraction, we decided to carry out a fractionation followed by a purification of the major compounds of this fraction. We first carried out a flash chromatography on a reverse phase, affording six subfractions. Purification of one of the fractions, using Biobeads SX-3 (BioRad, Hercules, CA, USA), furnished two pure compounds: rugosin D and tellimagrandin II.

In order to evaluate the XO inhibitory activity of the most promising compounds of the crude extract, we envisaged to measure the IC_50_ values of kaempferol, quercetin, rugosin D, and spiraeoside, as well as, presumably, inactive ones (gallic acid, hyperoside, isoquerctitrin, and salicylic acid) compared to allopurinol. As we could expect, phenol derivatives (gallic acid and salicylic acid) are not potent XO inhibitors, with IC_50_ values higher than 50 µM (Table 2). However, as previously described, rugosin D exhibits a rather strong inhibitory activity, with an IC_50_ of 35.7 ± 2.1 µM. The inhibitory activities of ellagic acid and tellimagradin II were not evaluated, as ellagic acid was not soluble in water or DMSO and tellimagradin II seemed to decomposed in the solution.

Nonglycosylated flavonoids, such as quercetin and kaempferol, exhibit strong XO inhibitory activity, with IC_50_ values of 3.5 ± 0.2 µM and 12.9 ± 0.6 µM, respectively, as described in previous works. Moreover, 3-*O*-glycosylated flavonoids, such as hyperoside and isoquercitrin, possess a weak inhibition rate, with an IC_50_ value above 50 µM (in comparison with allopurinol, with an IC_50_ of 17.2 ± 0.8 µM), in agreement with the literature [23].

More surprisingly, spiraeoside, a 4′-*O*-glycosylated quercetin derivative, exhibits very strong inhibitory activity, with an IC_50_ of 0.66 ± 0.02 µM. This compound seems to be more effective than allopurinol or quercetin themselves. A recent study confirms the high inhibitory activity of spiraeoside [24].

According to previous structure–activity relationship studies on the XO inhibitory activities of flavonoids, it has been demonstrated that glycosylation, at positions 3 and 7, decreases binding affinity compared to the native flavonoid. This phenomenon has been observed for quercetin, kaempferol and naringenin [15,25]. The results obtained for hyperoside and isoquercitrin, quercetin derivatives glycosylated in position 3, are in agreement with these studies. However, spiraeoside, a 4′-glycosilated quercetin derivative, seems to exhibit a strong affinity with the enzyme. The position of substitution on the flavonoid derivative seems to be a key factor for binding in the active site of the XO, as spiraeoside possesses an IC_50_ value five times lower than quercetin. The same results have been described for luteolin and luteolin-4′-*O*-glucoside [26]. These results also demonstrated that three compounds present in *F. ulmaria* aerial parts, kaempferol, quercetin, and spiraeoside, possess a stronger XO inhibitory activity than allopurinol.

The inhibitory activity of the most active compounds present in the extract of *F. ulmaria* having been identified, we developed complementary quick end efficient methods, either to facilitate the bioguided fractionation or to determine the active compounds in the crude extract: a strategy based on the evaluation of the interactions of the compounds present in the extract with the enzyme by HPLC analyses, followed by an HPTLC bioautography assay.

### 2.3. Investigation of Ethyl Acetate Fraction through HPTLC XO Bioautography

We first envisaged, in order to validate the HPTLC bioautography method, to perform the EDA strategy on the EtOAc fraction and the six subfractions obtained by flash chromatography, as we had already evaluated the XO inhibition activity of the major compounds present in these fractions.

As the spectrophotometer analysis monitors the production of uric acid from xanthine, the HPTLC method measures the production of the superoxide radical anion formed during the regeneration of an enzyme. In contact with superoxide radical anion, the Nitro Blue Tetrazolium (NBT) is transformed in formazan (purple), inducing a change in the color of the reaction mixture. The inhibitors will appear as white/yellow spots under a purple background.

**Figure 2:**
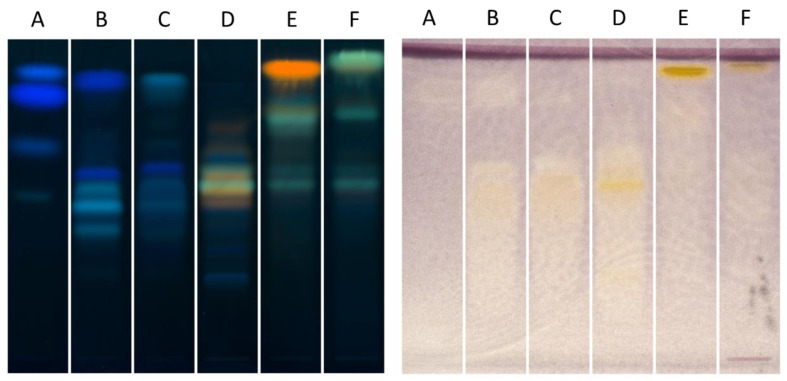
HPTLC analyses with NP-PEG derivatization (**left**) and XO bioautography (**right**) of fractions A to F.

In order to compare the phytochemical profile and the XO inhibition, we performed two HPTLC plates (Figure 2). The left plate (NP-PEG) shows that polyphenols, especially tannins (in pale or dark blue on the plate) are the major constituents of fractions A, B and C, as fractions D, E and F seem to contain mostly flavonoids, especially in fractions E and F. After comparison with the standards, we demonstrated that fraction E contains mostly quercetin (yellow spot at Rf 0.9) and fraction F contains mostly kaempferol (green spot at Rf 0.95).

A comparison of the two plates (Figure 2) seems to show that the polyphenols present in fractions A, B and C do not possess XO inhibitory activities, as flavonoids (major compounds of fractions D, E and F) possess high inhibitory activities. Indeed, spiraeoside (Rf 0.6, fraction D), quercetin, and kaempferol (fractions E and F) turn out to be quite strong inhibitors.

To confirm the results obtained by HPTLC, we determined the IC_50_ of each fraction using a UV method. If fractions A, B and C showed weak activity (IC_50_ > 100 µg/mL, Supplementary Material Figure S2), fraction D and F exhibited a good activity, with IC_50_ values of 2.3 ± 0.1 µg/mL an IC_50_ of 3.4 ± 1.4 µg/mL, respectively. Fraction E was the most active fraction, with an IC_50_ of 1.33 ± 0.03 µg/mL. We confirmed that an HPTLC autobiography could be a quick and efficient method to evaluate the XO inhibitory activity of plant extracts but, also, of pure compounds.

The next step of our study consisted of using an HPTLC autobiography to identify potential XO inhibitors present in the bioactive fractions.

### 2.4. Determination of Active Compounds by HPTLC Bioautography

**Figure 3:**
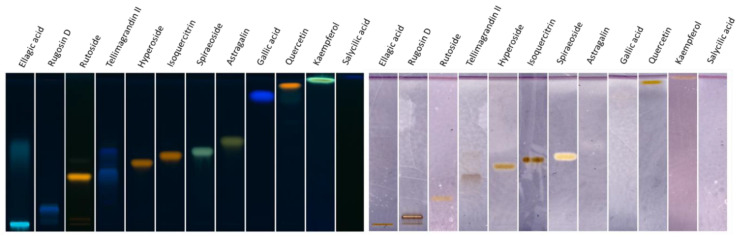
HPTLC analyses with NP-PEG derivatization (**left**) and XO bioautography (**right**) of 12 pure compounds.

In order to find the more active XO inhibitors and to validate our method, we chose to perform the HPTLC analyses on twelve pure compounds (Figure 3), particularly flavonoid derivatives and tannins. Among these compounds, we envisaged to test salicylic acid and gallic acid, described to possess no inhibitory activity (IC_50_ > 100 µM) [27,28] for a “negative assay”.

As described above, five compounds seemed to show no significant activity (Figure 3)—gallic acid and salicylic acid, as we could expect, but also rutoside and astragalin, known to be very weak inhibitors. However, tellimagrandin II and ellagic acid seem to decompose on the plate, which could explain their weak activity.

The spots obtained for other compounds should indicate that they exhibit interactions with the enzyme. The intensity of the spots for rugosin D, quercetin, kaempferol and spiraeoside confirms the strong interactions of these compounds with XO, as hyperoside and isoquercitrin exhibit weak interactions.

We demonstrated that an EDA strategy is a quick and efficient method, either to perform bioguided fractionation or to evaluate the XO inhibitory activity of secondary metabolites.

To complete this strategy, we envisaged to develop an HPLC method that could allow the determination of compounds able to link with enzymes in complex mixtures.

### 2.5. Detection of Potential Inhibitors by HPLC Analysis

**Figure 4:**
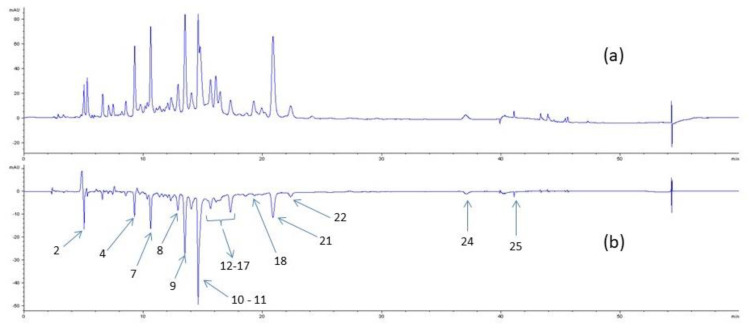
HPLC-UV 254 nm profiles of *F. ulmaria* aerial parts methanolic extract: (**a**) crude extract; (**b**) difference of the chromatogram of the crude extract without and with the enzyme (negative peaks indicate interaction).

The identification of bioactive compounds which form in a complex mixture by rapid screening, without the requirement of purification, is a challenging approach. Techniques based on HPLC analyses of the crude extract, after the determination of the phytochemical profile and after incubation with the desired enzyme, could allow the identification of individual active compounds in the mixture. Based on the method described by Wang [29], we incubated the enzyme with the crude extract and carried out HPLC analyses on the filtrate after centrifugation. The method used in this study does not involve enzyme ultrafiltration but a simple filtration in order to remove the enzyme from the reaction mixture. Subtraction of the chromatogram of the crude extract before and after incubation with XO allowed the detection of the compounds able to interact with the enzyme (Figure 4), as they appear as negative peaks. The inactive ones were not present on the subtracted spectra (b). This method is efficient for quickly evaluating the interactions of secondary metabolites with the enzyme. However, it does not permit a determination of the inhibitory activity of the compounds present in the crude extract. Indeed, the potent interactions of compounds with XO in a crude extract could be related to their IC_50_ values and, then, with its potential inhibitory activity. Nevertheless, this method is useful for performing bioguided fractionation to find out potential enzyme inhibitors.

The chromatograms (Figure 4) showed that around ten compounds seemed to interact with XO, as the area of the corresponding peak is lower in the presence of the enzyme. The most potent results were obtained for ellagitannins, such as rugosins and tellimagrandins, as well as for ellagic acid. Rugosin D and tellimagrandin II seem to present the strongest interaction with XO. These results are in agreement with previous studies and the IC_50_ values measured for rugosin D, quercetin and spiraeoside, showing that tannins are rather good XO inhibitors, especially concerning rugosin D and tellimagrandin II, which have quite similar IC_50_ values [30].

**Figure 5:**
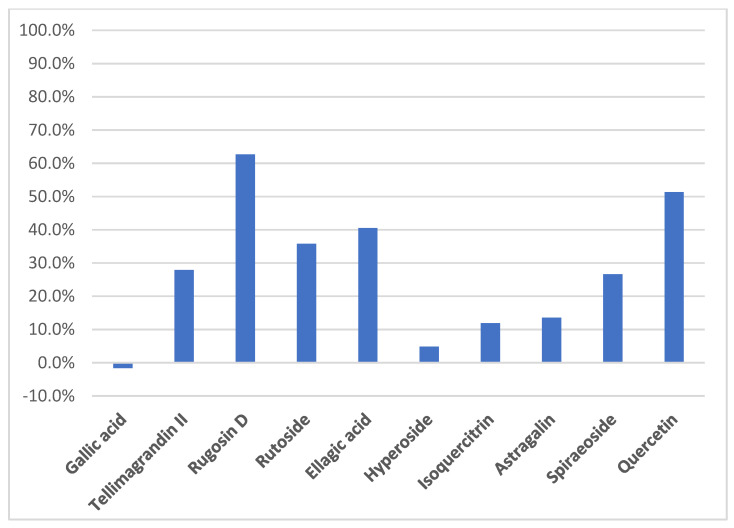
Evaluation of XO binding (%) performed by HPLC analyses.

Regarding the difference of area between the peak of each compound with and without the presence of XO, we could estimate the percentage of binding for each compound with the enzyme (Figure 5). Rugosin D and quercetin seem to bind with strong interactions with the enzyme, with around 60% and 50% of binding, respectively. Considering rutoside, the percentage of binding is probably overvalued because several compounds probably have the same retention time. With around 30% of binding, tellimagrandin II and spiraeoside could be potential XO inhibitors.

We demonstrated that flavonoids, such as quercetin or kaempferol, seem to interact with the enzyme. More surprisingly, among glycosylated flavonoids, only spiraeoside (quercetin-4′-*O*-glucoside) and kaempferol-4′-*O*-glucoside showed significant interactions with the enzyme, whereas isoquercitrin or hyperoside did not seem to interact with XO. Nevertheless, previous studies described that dietary flavonoids, such as quercetin, kaempferol or luteolin, are better inhibitors than glycosylated flavonoids, such as hyperoside or astragalin.

The HPLC method seems to be efficient for identifying the compounds able to bind to an enzyme in a complex matrix. This strategy could be useful for bioanalytical assays to perform bioguided fractionation on a complex matrix, particularly plant extracts.

## 3. Conclusions

Based on its traditional uses, particularly for gout disease, we investigated the chemical profile of *F. ulmaria* and evaluated either the XO inhibition activity of the aerial parts extract or the XO inhibition activity of some isolated compounds present in the mixture. As the methanolic aerial parts extract of *F. ulmaria* exhibits strong inhibitory activity, with an IC_50_ value of 8.3 ± 0.3 µg/mL, we performed a fractionation of the crude extract and further partitioned the chromatography of some fractions by RP-18 silica gel. Then, we determined the XO inhibition activity by measuring the IC_50_ values of some secondary metabolites. As previously described, we demonstrated that flavonoids, such as quercetin and kaempferol, glycosylated flavonoids, such as spiraeoside, and tanins, such as rugosin D, are strong XO inhibitors. We confirmed that quercetin and kaempferol exhibit high activity, whereas 3-*O*-glucosyated quercetin derivatives, such as hyperoside and isoquercitrin, did not seem to interact with the enzyme. Nevertheless, spiraeoside, a 4′-*O*-glycosilated quercetin derivative, seems to be a strong inhibitor. We supposed that the activity of *F. ulmaria* against gout disease could be due to the presence of spiraeoside, which is known to be one of the major constituents present in the flowers and leaves of *F. ulmaria* [20] and possesses strong XO inhibitory activities.

In order to facilitate the screening of potential enzyme inhibitors, we developed two quick and efficient analytical methods: HPLC analyses and an HPTLC autobiography. To facilitate the bioguided fractionation of such inhibitors, we have developed an efficient HPLC method, allowing us to determine compounds exhibiting interactions with XO. This method is quite efficient for either evaluating the active fractions in a bioguided fractionation strategy or for determining the inhibitory activity of isolated compounds. The HPLC method developed allows the evaluation of compounds exhibiting interactions with an enzyme, and we demonstrated that the percentage of binding, easily determined, is directly correlated with inhibitory activity. This strategy could be useful either to reveal the compounds potentially active in a complex matrix or to evaluate the capacity of inhibition of compounds, as this method could be quantitative.

## 4. Materials and Methods

### 4.1. Plant Material

The aerial parts of *F. ulmaria* were collected in Saint-Genès Champanelle (France) in July 2019 and identified by Arnaud Delcoigne from the Université Clermont-Auvergne herbarium (Clermont-Ferrand, France). A voucher specimen was deposited at the herbarium (CLF121184).

### 4.2. Standards and Reagents

All chemical products and reagents were purchased from Sigma-Aldrich (Saint-Louis, MO, USA). All chemical standards and references were purchased from Extrasynthese (Genay, France), Sigma-Aldrich or Carbosynth (Compton, United-Kingdom).

### 4.3. Extraction, Characterisation and Isolation

#### 4.3.1. Extraction of Aerial Parts of *F. ulmaria*

The aerial parts of *F. ulmaria* were air-dried at room temperature in the dark, powdered, and extracted three times with methanol for 24 h (75 g of powdered dried plants with 3 × 750 mL of methanol). After filtration, the methanol extract was then dried under vacuo to furnish a dry yellowish extract (crude extract, 22.3 g) in approximately a 30% yield.

#### 4.3.2. Characterization of the Crude Extracts

HPLC analyses were performed on an Agilent 1260 Infinity apparatus, with a DAD detector equipped with an Uptisphere C18-3 (250 × 4.6 mm, 5µm) column from Interchim (Montluçon, France). LC-MS analyses were carried out on an UHPLC Ultimate 3000 RSLC chain and an Orbitrap Q-Exactive (Thermo Scientific, Waltham, MA, USA) with the column mentioned above. Source operating conditions were as follows: 3 kV spray voltage; 320 °C heated capillary temperature; 400 °C auxiliary gas temperature; sheath, sweep and auxiliary gas (nitrogen) flow rate at 50, 10 and 2 arbitrary units, respectively; s collision cell was used in stepped nCE mode, with an ionisation voltage between 10 and 50 arbitrary units. Full scan data were obtained at a resolution of 70,000, whereas MS^2^ data were obtained at a resolution of 17,500. Data were processed using Xcalibur software (Thermo Fisher Scientific Inc., Waltham, MA, USA). The identification of all compounds described was carried out using the negative ionisation mode.

For both analyses, the mobile phase was a mixture formic acid in water (0.1% *v*/*v*) (phase A) and formic acid in acetonitrile (0.1% *v*/*v*) (phase B). The gradient of phase A was 100% (0 min), 80% (10 min), 73% (35 min), 0% (40–50 min), and 100% (51–60 min). The flow rate was 0.8 mL/min and the injection volume was 5 µL.

#### 4.3.3. Purification of Compounds from *F. ulmaria* Aerial Parts

The crude methanolic extract was dissolved in distilled water (400 mL for 10 g) and extracted three times with increasing polarity solvents (*n*-hexane, dichloromethane, ethyl acetate and butanol) to provide five fractions. The ratio between the aqueous phase and the organic layer was 4:1 (*v*/*v*).

Part of the ethyl acetate fraction (991 mg out of 1228 mg) was subjected to a flash chromatography to afford six fractions (A to F). A dry load of polar fractions on reversed phase silica gel was prepared and packed as a sample. The polar fraction was then submitted to flash chromatography through a Chromabond C18 (80 g) (Macherey-Nagel, Hoerdt, France) column with water—acetonitrile gradient at 45 mL/min. Detection of compounds was performed at 254 nm. The mobile phase was a mixture of water (phase A) and acetonitrile (phase B). The gradient of phase A was 100% (0–5 min), 0% (55 min), and 0% (55–60 min).

Fraction D was then partitioned with Biobeads SX-3 column chromatography (BioRad, Hercules, CA, USA) in THF to afford rugosin D (10.4 mg) and tellimagrandin II (11.7 mg). Rugosin D and tellimagrandin II were identified by NMR using an Avance III HD 500 MHz spectrometer (Bruker, Billerica, MA, USA) with CD_3_OD as solvent.

### 4.4. Xanthine Oxidase Inhibitory Activity

Inhibition of XO was conducted according to the procedure described by Sowndhararajan et al. [23], with slight modifications. The assay mixture contained 770 µL of 120 mM PBS, 700 µL of xanthine solution in PBS (final concentration of 56 µM), 490 µL of plant extract in PBS (range of final concentration from 10 to 100 µg/mL), and 140 µL of XO solution (final concentration of 0.01 U/mL in PBS). Prior to the addition of XO solution in the mixture, all the components were incubated at 25 °C in the dark for 5 min. The reaction was initiated by adding the XO solution and the evolution of absorption is measured at 293 nm, indicating the formation of uric acid, for 5 min. The enzymatic activity assay without extract was defined as maximum relative activity, and the percentage of inhibition was calculated according to the method of Yan et al. [31] IC_50_ values were determined using linear regression and are expressed in µg/mL of plant extract ± standard deviation. All measurements were performed in triplicate (*n* = 3).

### 4.5. HPTLC Analysis

#### 4.5.1. Equipment

Materials included a CAMAG HPTLC system (Muttenz, Switzerland) equipped with an Automatic TLC Sampler (ATS 4), an Automatic Developing Chamber (ADC2) with humidity control, a TLC Visualizer, VisionCATS software and a Chromatogram Immersion Device III, and TLC Plate Heater III for derivatization.

#### 4.5.2. General Procedure

The samples were prepared by dissolving 1 mg of each fraction in 1 mL of methanol, and 20 µL was applied on 8 mm bands, 8 mm from the lower edge of the plate. The mobile phase was a mixture of ethyl acetate, dichloromethane, formic acid, acetic acid, and water (100:25:10:10:11). Plates were developed over a distance of 70 mm from the lower edge, using a twin trough glass chamber saturated for 20 min with a mobile phase under controlled humidity (RH: 33%). After development, plates were dried under a stream of cool air for 10 min.

#### 4.5.3. Natural Products Reagent Derivatization

After elution, the plates were heated at 100 °C for 2 min and dipped into a solution of 2-aminoethyl diphenylborinate in ethyl acetate (30 mM). After taking a picture at 366 nm and white light, the plates were dipped into a solution of polyethylene glycol 400 in ethyl acetate (5% *v*/*v*), and a new picture was taken at 366 nm and white light.

#### 4.5.4. Xanthine Oxidase Bioautography

The XO inhibition assay was conducted using the method developed by Ramallo [32], with slight modifications. After migration, the plates were dipped into a solution of PBS 120 mM containing xanthine oxidase (0.1 U/mL), EDTA (1 mM), and NBT (1 mM) in a sterilizer, at 37 °C in the dark for 30 min. The plates were then dipped into a solution of PBS 120 mM containing xanthine (1.5 mM) in a sterilizer, at 37 °C for 30 min in the dark. Inhibitors appeared as white/yellow spots on a purple background.

#### 4.5.5. Measurement of XO Interaction Involving HPLC Method

The measurement of XO interaction was conducted according to Zhang’s [33] procedure, with some modifications. The assay mixture contained 10 mL of crude extract at 1 mg/mL in water, and 200 µL of xanthine oxidase (final concentration of 0.1 U/mL), or 200 µL of water. Prior to the HPLC analysis, the solution was mixed for one hour at room temperature in the dark. After one hour, the mixture was centrifuged at 4500× *g* RPM and supernatant was filtered through a 0.45 µm PTFE filter. Samples were then analyzed using an Agilent 1260 Infinity apparatus (Agilent, Santa Clara, CA, USA), with a DAD detector equipped with an Uptisphere C18-3 (250 × 4.6 mm, 5µm) column from Interchim, with the same method as mentioned above. Potential inhibitors were identified after subtraction of the chromatogram obtained from the assay with and without XO (negative peaks).

## Figures and Tables

**Table 1:** Compounds identified in *F. ulmaria* aerial part extract.

**N°**	Rt (min)	Compound	Formula	M − H_exp_ (m/z)	MS^2^ Fragment	Reference
**1**	3.84	Quinic acid	C_7_H_12_O_6_	191.0549	191/85/192/127/93	Standard
**2**	6.81	Citric acid	C_6_H_8_O_7_	191.0189	111/87/85/191/129	Standard
**3**	8.52	Gallic acid	C_7_H_6_O_5_	169.0131	125/169/126/170/97	Standard
**4**	12.97	Chlorogenic acid	C_16_H_18_O_9_	353.0882	191/353/85/161/179	Standard
**5**	12.73	Rugosin B	C_41_H_30_O_27_	953.0912	301/275/249/765/909	Bijttebier et al., 2016
**6**	13.10	Catechin	C_15_H_14_O_6_	289.0717	289/245/109/125/203	Standard
**7**	13.38	Tellimagrandin I	C_34_H_26_O_22_	785.0887	301/275/785/249/169	Bijttebier et al., 2016
**8**	14.48	Rugosin E	C_75_H_54_O_48_	860.0865^c^	301/275/169/249/785	Bijttebier et al., 2016
**9**	15.12	Rugosin A	C_48_H_34_O_31_	1105.1011	301/275/166/1061/937	Bijttebier et al., 2016
**10**	15.50	Tellimagrandin II	C_41_H_30_O_26_	937.0958	301/275/937/169/249	Bijttebier et al., 2016
**11**	15.86	Rugosin D	C_82_H_58_O_52_	936.0930^c^	301/169/275/451/767	Standard
**12**	17.01	Rutoside	C_27_H_30_O_16_	609.1459	300/609/301/271/255	Standard
**13**	17.88	Ellagic acid	C_14_H_6_O_8_	300.9987	301/302/229/257/283	Standard
**14**	18.16	Isoquercitrin	C_21_H_20_O_12_	463.0885	300/463/301/271/255	Standard
**15**	18,37	Quercetin-3-*O*-(2″-*O*-galloyl)-β-galactopyranoside	C_28_H_24_O_16_	615.0996	301/151/178/313/302	Bijttebier et al., 2016
**16**	18.58	Hyperoside	C_21_H_20_O_12_	463.0882	300/463/301/271/255	Standard
**17**	18.70	Miquelianin	C_21_H_18_O_13_	477.0677	301/477/151/179/255	Bijttebier et al., 2016
**18**	22.13	Astragalin	C_21_H_20_O_11_	447.093	284/447/285/151/107	Standard
**19**	22.78	Astragalin-2″-*O*-gallate	C_28_H_24_O_15_	599.1052	285/313/257/169/229	Chen et al., 2018; Samardžić et al., 2018
**20**	23.11	Isorhamnetin-*O*-hexoside	C_22_H_22_O_12_	477.1041	477/314/271/243/285	Bijttebier et al., 2016
**21**	23.86	Spiraeoside	C_21_H_20_O_12_	463.0880	301/151/300/463/178	Standard
**22**	25.38	Kaempferol-4′-*O*-glucoside	C_21_H_20_O_11_	447.0934	447/284/285/151/448	Bijttebier et al., 2016
**23**	30.72	Salicylic acid	C_7_H_6_O_3_	137.0229	93/137/94/138/65	Standard
**24**	38.83	Quercetin	C_15_H_10_O_7_	301.0351	301/151/179/121/107	Standard
**25**	40.46	Kaempferol	C_15_H_10_O_6_	285.0418	285/286/257/185/229	Standard

^a^ Identified with analytical standard; ^b^ identified according to the literature data; ^c^ [M − 2H]^2−^; in bold the fragmented mass.

**Table 2:** IC_50_ Values of purified compounds measured by UV spectroscopy.

Compound	IC_50_ (µg/mL)	IC_50_ (µM)
Allopurinol (control)	2.9 ± 0.1	17.2 ± 0.8
Gallic acid	>300	>50
Salicylic acid	>300	>50
Rugosin D	67.0 ± 1.1	35.7 ± 2.1
Kaempferol	3.7 ± 0.2	12.9 ± 0.6
Quercetin	1.07 ± 0.06	3.5 ± 0.2
Hyperoside	>100	>50
Isoquercitrin	>100	>50
Spiraeoside	0.31 ± 0.01	0.66 ± 0.02

## Data Availability

Not applicable.

## Supplementary Materials

Supplementary Materials are available online. Figure S1: XO inhibitory activity of crude extract and fractions, Figure S2: Xo inhibitory activity of ethyl acetate fraction and sub-fractions A to F.
