# Supplementary material for: Xanthine Oxidase Inhibitors from Filipendula ulmaria (L.) Maxim. and Their Efficient Detections by HPTLC and HPLC Analyses
Source: Molecules. 2021 Mar 30;26(7):1939. doi: 10.3390/molecules26071939 (PMC8038090; doi:10.3390/molecules26071939)
Supplement: Supplementary file 1 [file molecules-26-01939-s001.pdf]

Supplementary material

Xanthine oxidase inhibitors from *Filipendula ulmaria* (L.) Maxim. and their efficient detections by HPTLC and HPLC analyses.

Maël Gainche [a]*, Clémence Ogeron [b], Isabelle Ripoche [a], François Senejoux [b], Juliette Cholet [b], Caroline Decombat [b], Laetitia Delort [b], Jean-Yves Berthon [c], Etienne Saunier [d], Florence Caldefie-Chezet [b] and Pierre Chalard [a]

[a] Université Clermont-Auvergne, CNRS, SIGMA Clermont, ICCF, F-63000 Clermont-Ferrand, France.

[b] Université Clermont-Auvergne, INRA, UNH, Unité de Nutrition Humaine, CRNH Auvergne, F-63000 Clermont-Ferrand, France

[c] Greentech, Biopôle Clermont-Limagne, 63360 Saint-Beauzire,

[d] Dômes Pharma, 3 Rue André Citroën, 63430 Pont-du-Château, France,

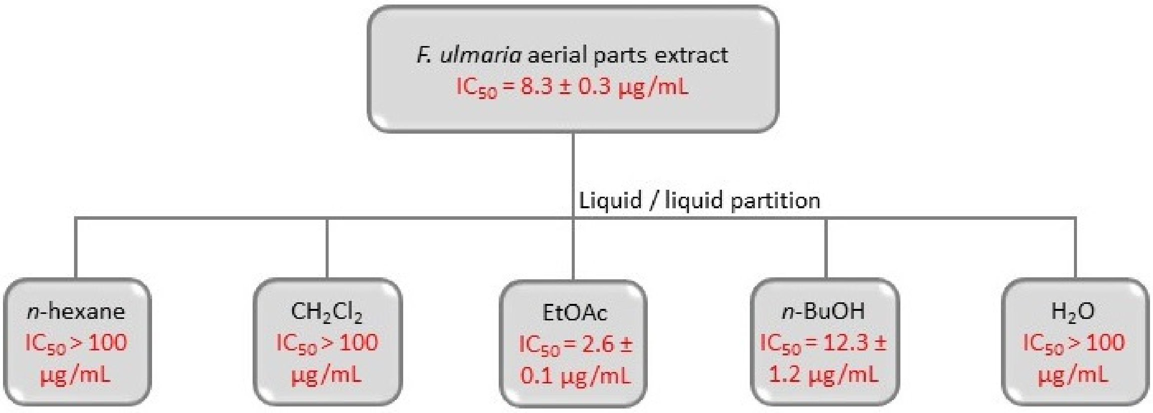

Figure S1 XO inhibitory activity of crude extract and fractions

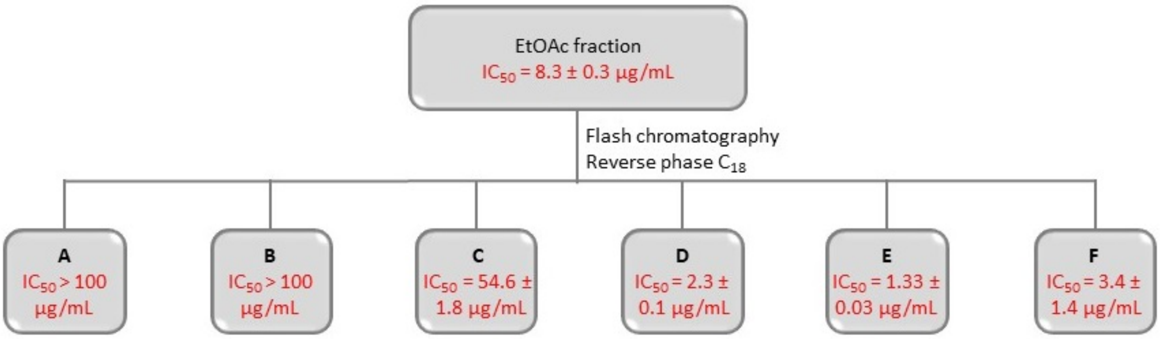

Figure S2 XO inhibitory activity of ethyl acetate fraction and sub-fractions A to F
